# TOXTRUST: a tool leveraging the Dempster-Shafer Theory for robust integration of NAM results in decision-making considering uncertainty

**DOI:** 10.1016/j.namjnl.2025.100043

**Published:** 2025-08-10

**Authors:** Karolina Kopańska, Adrian Cabrera, Manuel Pastor

**Affiliations:** a Research Programme on Biomedical Informatics (GRIB), Department of Medicine and Life Sciences, Universitat Pompeu Fabra, Hospital del Mar Research Institute, Barcelona, Spain; b Center for Alternatives to Animal Testing (CAAT), Johns Hopkins University, Baltimore, USA

**Keywords:** Dempster-Shafer Theory, Uncertainty, Evidence Combination, New Approach Methodologies, Next Generation Risk Assessment

## Abstract

In the search for alternatives to replace *in vivo* studies, the application of assessment frameworks involving New Approach Methodologies (NAMs) often leads to the simultaneous availability of multiple pieces of evidence of different quality. When integrated into defined data structures, combinations of NAMs can generate answers to complex toxicological questions. However, if they are not integrated correctly, a collection of NAM results may produce misleading results that complicate the assessment process or lead to wrong conclusions.

To support transparent decision-making in situations in which multiple NAMs are applied to generate results for the same toxicological question, we developed TOXTRUST (www.github.com/phi-grib/TOXTRUST) — an open-source computational tool integrating the mathematical framework of the Dempster-Shafer Theory (DST).

In this article, we briefly describe the DST framework for the integration of NAM results to define the scope of its application and data requirements. This is followed by a description of the functionalities and infrastructure of TOXTRUST. Lastly, we illustrate how TOXTRUST can be applied to any endpoint with binary end-results, with a focus on the generation and interpretation of results expressed through probability bounds.

## 1 Introduction

Toxicological risk assessment is a multi-stage process of testing and analysis aimed at the evaluation of the safety of a product, considering its composition, exposure scenarios, and associated hazards. For decades, toxicological studies were conducted nearly solely through *in vivo* experiments. Recently, extensive efforts were undertaken to move towards the Next Generation Risk Assessment (NGRA), defined as an exposure-led and hypothesis-driven approach that integrates results derived from the New Approach Methodologies (NAMs) (Dent et al. 2021; Carmichael et al. 2022).

Given the human-relevant design of NAMs, their adoption naturally resolves some translational issues and supports the 3Rs principles (Replacement, Reduction, and Refinement) in toxicological experimentation (Brescia et al. 2023). However, the downside of replacing whole-animal *in vivo* studies with NAMs is their limited biological coverage focused on particular mechanisms of toxicity (Escher et al. 2022). Therefore, when alternative systems are used to test specific NGRA hypotheses, one implicit task is to combine multiple pieces of evidence into a “battery” that covers a “biological domain” (Sewell et al. 2024). However, in today’s data- and method-rich environment multiple NAMs may be used to target the same mechanism, and therefore, the second crucial task is to integrate data addressing the same endpoint accurately.

For many years, pieces of toxicological evidence have been mainly combined by experts “in the brain” based on their judgment or through the application of universal rules such as voting or weighting (Hardy et al. 2017). Despite their extensive use, such approaches tend to oversimplify the process of data integration and may therefore not be adequate to handle complex systems based on NAMs. Moreover, experimental, or computational results and scientific decisions based on their combination are always associated with some degree of uncertainty (Suciu et al. 2023), which people generally struggle to estimate correctly (Paparella et al. 2013). Due to these reasons, expert judgments are known to be to some extent subjective, often leading to poor reproducibility of the procedure and hence, of the final decisions.

**Fig. 1:**
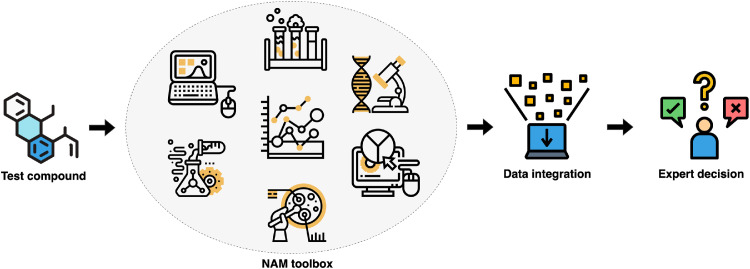
The NGRA, which integrates a series of NAMs, yields data that vary in quality, relevance to the endpoints of interest, and the levels of associated uncertainty. This often poses a difficulty for experts who simultaneously consider multiple pieces of diverging information to derive assessment conclusions.

This problem is schematically represented in Fig. 1, showing that the integration of data, which is parallelly derived by applying different NAMs to the test chemicals, poses a substantial problem for experts. This situation is worsened by the widely acknowledged fact that human beings have limited skills for making objective decisions when multiple elements of information, of diverse origins and quality, must be considered simultaneously (O’Hagan 2019).

Nowadays, NAMs are increasingly incorporated into Integrated Testing Strategies (ITS), Integrated Approaches to Testing and Assessment (IATA), Defined Approaches (DA) and NGRA workflows, where they are systematically combined. However, it has been argued that these strategies should be supplemented with probabilistic approaches to better integrate NAM-derived results, thereby accounting for uncertainty (Maertens et al. 2022).

While the traditional strategies for probability are the most applied in toxicological decision analysis, there are several alternatives (Dubois et al., 2011). Among all alternative mathematical approaches that allow considering several lines of partially uncertain evidence to come to a decision, the Dempster-Shafer Theory (DST) stands out as the one with the highest degree of theoretical development (Sentz and Ferson, 2002; Shafer, 2016).

The Dempster-Shafer Theory evolved from the work of Arthur P. Dempster (Dempster 1967a; 1968) and was further developed and described by Glenn Shafer as “A mathematical theory of evidence” (Shafer 1976), alternatively referred to as the Theory of Belief Functions. The DST provides a robust framework for combining probabilities from multiple independent observations of the same event under the assumption that their integration leads to a more reasonable conclusion (Dempster 2008). Combination results are expressed as Bounded Probability, ranging between the degrees of Belief and Plausibility, which facilitates a realistic interpretation of the conclusion and the associated uncertainty.

In the context of toxicology, Park et al. (2014) described the application of the DST for combining risk estimates derived from scientific conclusions and Rathman et al. (2018) described its use for combining *in silico* predictions. While the DST is seen as a promising method for toxicological decision-making (Moné et al. 2020), there is one missing component: a user-friendly digital solution for easy application. Indeed, to increase the true practical value of probabilistic methods, like the DST, it is essential to develop intuitive computational tools that would guide users through the application process. Risk assessment is complex, and handling mathematical or statistical methods can be challenging, so experts need accessible tools that clearly outline both, their applicability and limitations.

In this article, we first describe the DST framework for the integration of NAM results to define the scope of its application and data requirements. Then, we present TOXTRUST, a user-friendly computational tool that applies the DST formalism to toxicological problems and translates experimental and computational results into mathematically grounded and transparent decisions that account for uncertainty. Later, we illustrate how TOXTRUST can be applied to any endpoint with binary end-results, with a focus on the generation and interpretation of results expressed through probability bounds.

## 2 Mathematical framework of the Dempster-Shafer Theory

### 2.1 Choosing an appropriate framework

Toxicological questions can often be addressed through different interpretations of probability, each with its advantages and disadvantages. Hence, the choice of the best strategy should be guided by the type of the problem and the characteristics of the available evidence (Aven and Reniers 2013).

Among the arguments in favour of the DST, especially in comparison with classical Bayesian methods, is the possibility of representing partly uncertain or incomplete evidence without requesting precise prior probabilities (Shafer 1986). Instead, the flexible DST framework derives probabilities for all possible answers to a question, including the answer "I don’t know", by merging the results generated by the given sources of evidence with the information on their reliability. In DST, the representation of uncertainty through the degree of Belief and Plausibility is more abstract yet provides a more detailed range of uncertainty than the simpler interval-based approach of Imprecise Probability Theory (Caselton and Luo 1992). Also, this framework allows the application of probabilistic methods when a relevant probabilistic model for the outcome, required by the Frequentist approach, is lacking (Shafer 1986). In contrast to other alternatives like Fuzzy Logic (Zadeh 1988), DST offers clear rules for integrating evidence from various sources into a mathematically valid result, which significantly enhances its practical applicability. Evidence integration is also possible even with conflicting or weak evidence, which can further aid in resolving complex toxicological issues.

Regarding its limitations, first and foremost, if multiple evidence pieces are to be combined, they must be connected through the same question. In other words, the DST cannot be applied in situations in which the considered data relate to different questions (e.g., the previously mentioned “battery” that covers a “biological domain”). Second, due to its foundation in Set Theory, the DST is not designed to handle quantitative data and can therefore only be used to address binary classification (e.g.: “toxic” and “non-toxic” classes) or categorical (e.g.: “mild”, “intermediate”, and “high” toxicity grades) problems. Third, ensuring full independence of evidence pieces may sometimes be challenging given the complexity behind toxicological testing.

### 2.2 Independence of evidence

The aspect of independence, consisting of three main conditions, is fundamental to ensure the mathematical validity of combination results produced using the DST framework. For a comprehensive mathematical description of the most relevant concepts, we refer to the article by Couso and Moral (2010).

The first condition requires that the occurrence of any considered event is independent of the occurrence of another considered event. In the context of the NGRA, the statistical term “event” can be interpreted as the result produced by a NAM. Under this definition, the first condition of independence is fulfilled when the result generated by the given NAM does not depend on or affect the result produced by another NAM. Adverse Outcome Pathways (AOPs), for instance, generally fail to fulfil this requirement since the occurrence of Key Events inevitably depends on the occurrence of the Molecular Initiating Event. Consequently, the DST does not provide appropriate methods to combine probabilities collected for different events along the AOP.

The second condition refers to the methodology and implies that the NAMs that generate separate evidence pieces meant to be combined for the same endpoint are independent. This condition is automatically met when the testing methods are of different types, for instance, *in vitro* and *in silico*. However, in cases when the same method is applied twice, some additional criteria must be fulfilled. To give an example, we propose considering two QSAR models as independent if their training series do not share compounds, and the molecular descriptors and/or the machine-learning algorithms are sufficiently different.

The third condition imposes that the procedures involved in selecting different evidence pieces are independent. Simply put, when a NAM generates a negative result, we cannot adjust the testing pipeline and “cherry-pick” a particular NAM, from which we expect the highest probability of generating a second negative result.

### 2.3 Handling toxicological evidence

**Fig. 2:**
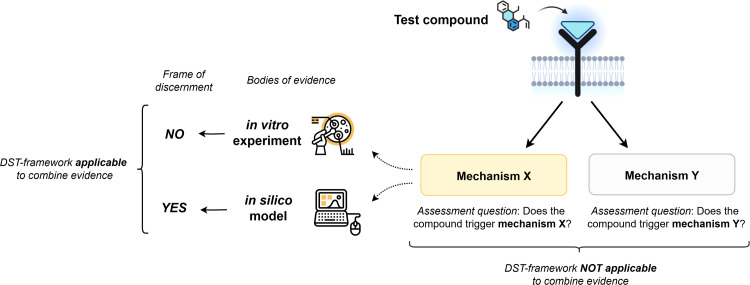
In toxicology, the DST framework can be applied to combine pieces of evidence that point to the same question (e.g., results from an *in vitro* experiment and an *in silico* model answering the question “Does the compound trigger mechanism X?”). This framework is not applicable to combine evidence pointing to different questions (e.g., covering different mechanisms of toxicity).

The practical application of the DST in toxicology, exemplarily illustrated in Fig. 2, requires three elements: a clearly defined ***assessment question***, a ***frame of discernment***, and one or more relevant ***bodies of evidence***.

The assessment question must be limited to a single toxicological property or safety endpoint, for instance, *“Does the compound trigger mechanism X?”.* The frame of discernment is a universal set containing a collection of all possible answers to the given question. Intuitively, the frame of discernment contains two elements: “No” (“Negative”) and “Yes” (“Positive”). The bodies of evidence are formalised data sources, using which probability measures are derived for the possible answers to the assessment question. Testing within the NAM domain could, for instance, involve a specific *in vitro* experiment and an *in silico* model, both qualitatively modelling the mechanism X. Consequently, the DST framework could be applied to combine results generated by these bodies of evidence.

### 2.4 Representation of uncertainty

The frame of discernment allows the construction of the ***universal set*** X, which contains all possible answers to the assessment question, as shown in example *(1)*.

(1) X={Negative,Positive}

However, in DST, probabilities are not only assigned to the elements from the universal set but also to their intersections (Sentz and Ferson 2002). These intersections are comprised within a ***power set***, which can be seen as a container for all possible subsets (*2^n^*) of the universal set, with *n* being the number of elements in that universal set. In our example, the power set *P(X)* of the universal set X contains four elements (2^2^ = 4), as shown in *(2),* including the empty set Ø, the sets containing the exclusive outcomes “*Negative”* and *“Positive”*, and their intersection.

(2) P(X)={Ø,{Negative},{Positive},{Negative,Positive}}

**Fig. 3:**
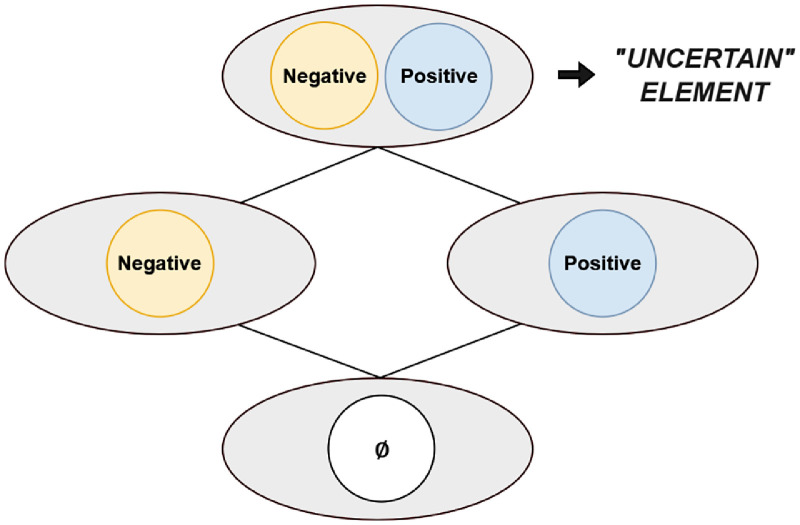
Representation of the interconnections between different elements of the power set *P(X)*, originating from the universal set X referring to an assessment question with binary end-results. The “Negative” and the “Positive” subsets are mutually exclusive since there are no logical commonalities between them. The conjoint subset can be seen as the “Uncertain” element since it allows for a situation in which given evidence is too weak to decide which of the exclusive elements of the universal set is the true outcome.

The logical connections between the different elements of the power set *P(X)* are illustrated in Fig. 3**.**

Both, the “Negative” and the “Positive” subsets are connected to the empty set because either outcome is possible before the experiment is physically conducted. In this context, the empty set can be viewed as a placeholder representing the absence of evidence. Once the experimental procedure is completed, the evidence source points to one of the possible outcomes. Moreover, these subsets are exclusive since there are no commonalities between them (A “Positive” outcome cannot be “Negative” at the same time). The conjoint subset, containing both answers, can be seen as the “Uncertain” element since it allows for a situation in which given evidence is too weak to decide which of the exclusive elements of the universal set is the true outcome (Beynon et al. 2001). Allowing for partial ignorance, the probability associated with the “Uncertain” element is alternatively referred to as the ignorance level.

### 2.5 Basic probability masses

Within the DST framework, elements of the power set are mapped to values in the interval between 0 and 1 considering the information provided by the bodies of evidence, employing the basic probability assignment function. The results of such mapping are referred to as ***basic probability masses*** *(m).* For individual mapping of every single element of the power set, the basic probability assignment function requires both the outcome generated by a NAM (e.g., “Negative” result) and the overall performance of the NAM in generating that outcome (e.g., “Specificity” value). These data points are integrated, yielding *m* values for each of the non-zero elements from the power set (“Negative”, “Uncertain”, “Positive”).

### 2.6 Ground probability masses

Mathematically, the combination of evidence requires a previous computation of basic probability masses for each element of the power set, and each considered body of evidence separately. Then, for any of those elements excluding the empty set, the basic probability masses are joined into a single numerical value, named ***ground probability mass*** *(q)*. Ground probability masses are the basis for evidence combination and were first introduced by Yager (1987). For a particular power set element, the joint structure *q* is a mathematically valid union of probability measures stemming from independent sources of evidence. The concept of ground probability masses is flexible and allows for updating the *q,* once a new body of evidence becomes available (e.g., a new NAM is developed for the measurement of a particular toxicological property).

### 2.7 Joint basic probability masses

The ground probability masses constitute separate inputs for combination rules; a special type of aggregation method (Shafer 1986). The rule-specific equations convert ground probability masses into the ***joint basic probability masses***. This conversion can be seen as the final step of “fusing” several probabilistic structures into a single probabilistic structure under the consideration of all available evidence pieces and their individual qualities. Hence, the joint basic probability masses are the probabilities that can be used to make decisions in toxicology.

### 2.8 Evidence combination rules

All DST-based combination rules differ from each other in terms of the estimation of uncertainty and the handling of conflicts. A comprehensive description of these differences was provided by Sentz and Ferson (2002) and Lepskiya (2013). In this manuscript and TOXTRUST, we respectively explain and implement the original ***Dempster’s rule, Yager’s modified Dempster’s rule***, and ***Inagaki’s unified combination rule***. These are the key combination rules that contributed to the establishment of the mathematical basis of the DST framework. For interested readers, novel literature offers insights into many other combination rules that build on them (Zhao et al. 2022; Su and Dong 2016; Dutta 2018; An et al. 2019; Sun and Wang 2018; Xia et al. 2018; Li and Fei 2019).

#### 2.8.1 Dempster’s rule

Dempster’s rule is critical to the original model of the theory and reveals the fundamental reasoning behind the combination of evidence (Dempster 1967; Shafer 2016). The Dempster’s rule is a strict “and” operation which disregards any disagreements between different bodies of evidence. This is achieved through a normalisation factor, calculated by taking the complement of the degree of conflict. However, since this rule is not appropriate to combine highly contradicting evidence, a general agreement between considered bodies of evidence must be established as the principal requirement for its application.

#### 2.8.2 Yager’s rule

Building on the original work of Dempster, Yager’s modified Dempster’s rule, shortly Yager’s rule, evolved a few years later (Yager 1987). In Yager’s rule, any disagreements between the considered bodies of evidence are regarded as coming from ignorance. Hence, Yager’s rule is the most suitable for the rigorous handling of uncertainty. Because of this rigorousness, however, this approach is less appropriate for pointing to mutually exclusive elements of the power set, since any conflicts are directly accounted for when computing the overall level of uncertainty.

#### 2.8.3 Inagaki’s rule

**Fig. 4:**
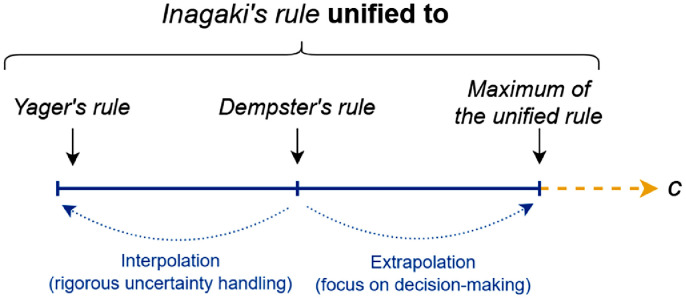
Graphical simplification of the interconnection between different combination rules via the non-negative normalization factor *c* described in Inagaki’s unified expression. This graphic was adapted from Inagaki (1991) and modified.

Inagaki’s unified combination rule was introduced to the scientific community in the context of nuclear plant safety (Inagaki 1991); however, the logic also applies to toxicological safety problems. In the original manuscript, Inagaki described that all valid combination rules can be unified using one equation that merges ground probability masses with the degree of conflict, the latter scaled with the non-negative parameter *c.* The reasoning behind this unified expression facilitates understanding how different combination rules address the conflict. This relationship is graphically shown in Fig. 4.

When Inagaki’s rule is applied, an accurate selection of parameter *c* is particularly important since it affects how conflicts are handled, and hence, how the uncertainty level in the combined result is computed. As *c* increases, the scaling effect on ignorance in the calculation becomes stronger. Hence, the highest value of *c* may facilitate decision-making as the effect of conflict is minimised. This, however, also brings about the possibility of introducing errors, since the uncertainty level may be inadequately low, and the assessment conclusion may fall on the wrong result.

### 2.9 The degrees of Belief and Plausibility

The real value of the DST lies in representing the results as bounded probability, rather than as single probability estimates. The lower and upper limits of such bounds are computed using the ***Belief*** function and the ***Plausibility*** function*,* respectively (Sentz and Ferson 2002; Rathman et al. 2018). These functions are applicable on both levels, including single evidence pieces or their combinations. The respective inputs are the basic probability masses or the joint basic probability masses.

**Fig. 5:**
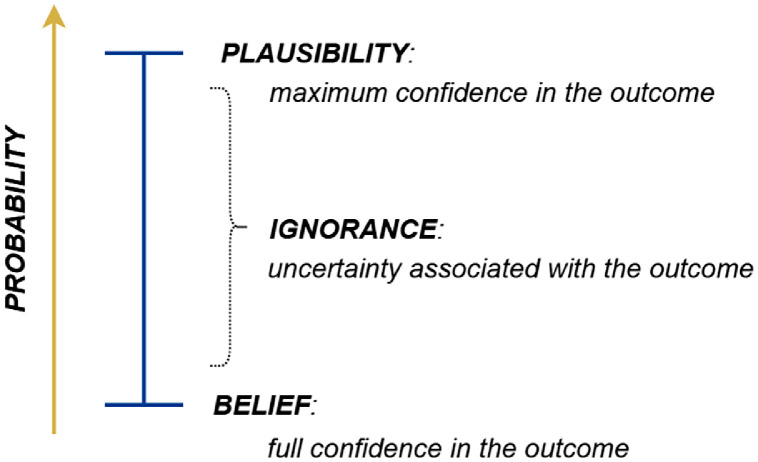
A bounded representation of the degree of Belief and Plausibility provides valuable insights into the level of uncertainty in scientific results and the implications for subsequent decisions.

While the term Belief corresponds to the probability that fully relates to a specific outcome, Plausibility is the maximum probability that can be attributed to it (Dempster 1967). To illustrate using our power set P(X), the Belief associated with a “Negative” outcome reflects the probability level at which we can have full confidence that the outcome is indeed “Negative.” On the other hand, the Plausibility of the “Negative” outcome represents the probabilistic overlap between the “Negative” element and other elements in the power set. For example, if the Belief and Plausibility values for the “Negative” outcome are 0.6 and 0.8, respectively, the difference between these values represents the “ignorance” or uncertainty mentioned above. As shown in Fig. 5, such bounded probability representation provides valuable insights for toxicologists, helping to significantly support and refine decision-making processes.

## 3 Methodological integration into TOXTRUST

To overcome several methodological challenges and help toxicologists apply the DST in praxis, we integrated the described mathematical framework into TOXTRUST; a user-friendly Python-based tool. TOXTRUST is an open-source software that can be redistributed or modified under the GNU General Public License terms as published by the Free Software Foundation version 3.

### 3.1 Architecture

**Fig. 6:**
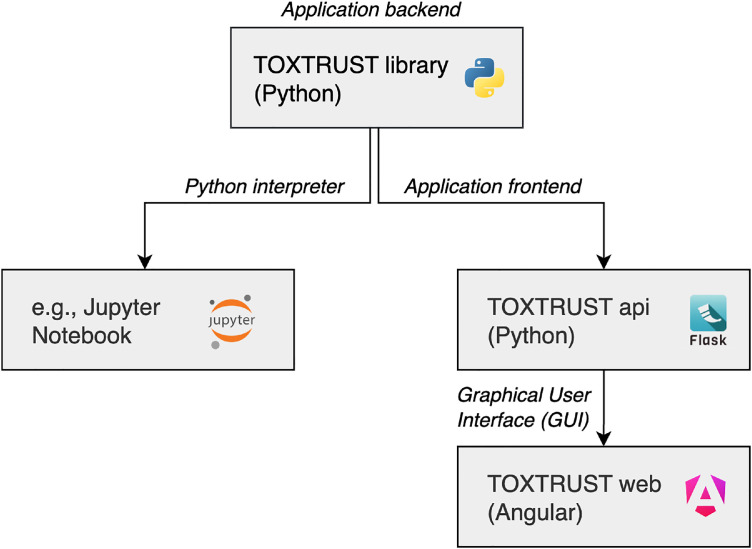
Architecture of TOXTRUST. The backend consists of a Python library which can be used in two different ways, either directly through a Python interpreter or a graphical user interface.

The architecture of TOXTRUST is illustrated in Fig. 6.

The backend of TOXTRUST consists of a Python library (https://github.com/phi-grib/TOXTRUST) which can be used in two different ways, either directly through a Python interpreter (e.g., Jupyter notebook) or a graphical user interface (GUI). TOXTRUST's backend and the GUI make use of Anaconda (Anaconda Software Distribution 2020) to define the essential libraries, facilitate their automatic installation in a private environment, and keep track of package versions to avoid incompatibilities. The code was written using Object Oriented Programming in Python 3.11, using four main packages including Pandas (McKinney 2011) version 1.5.3, NumPy (Harris et al. 2020) version 1.24.2, Matplotlib (Hunter 2007) version 3.7.0 and more-itertools (Rose and Bayles 2012) version 9.1.0. Before use, TOXTRUST must be installed within the Anaconda environment as a separate package, which can be used in development mode or user mode. TOXTRUST can be installed and used in Linux, Windows, and macOS operating systems. A concise description of the installation and setup process is available in the supplementary materials.

The backend is interconnected with the API (https://github.com/phi-grib/TOXTRUST_api) using Flask web framework (Grinberg 2018) version 3.0.3, which provides the tools and libraries needed to set up a server, define routes (URLs), and handle HTTP requests. In this setup, Flask runs on the backend as a server that receives incoming requests from the frontend, processes them, and returns appropriate responses.

The GUI (https://github.com/phi-grib/TOXTRUST_web) was built using Angular (Google 2016) version 18.0.6 which interacts with Flask by sending HTTP requests to interact with data through RESTful API endpoints. Flask processes these requests, performs the necessary backend operations, and returns JSON responses. Angular uses this data to dynamically update the GUI in real time. The GUI was designed to run locally as a desktop application, requiring the web server and the backend to operate on the same computer.

### 3.2 Main features

**Fig. 7:**
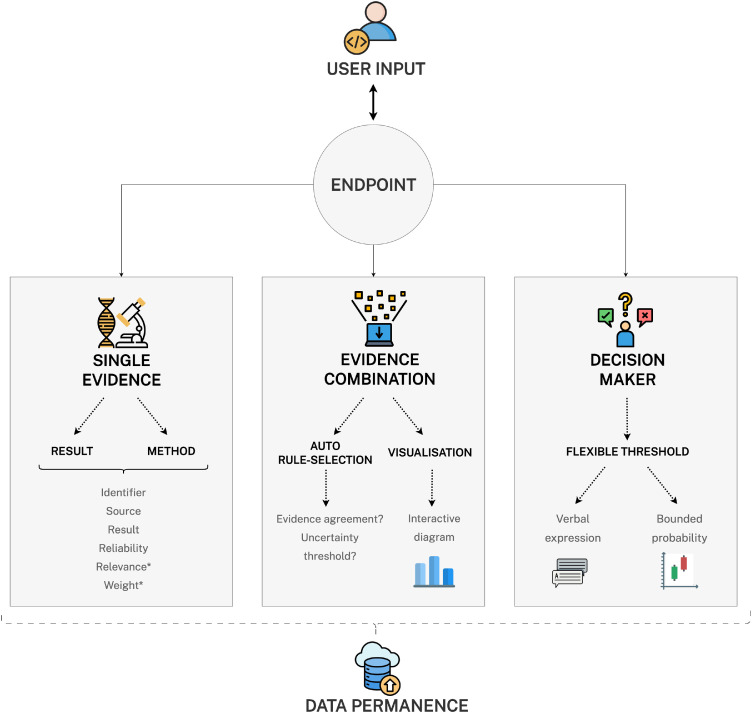
TOXTRUST's functionality is supported by three modules, each responsible for managing individual pieces of toxicological evidence, combining them, and making decisions based on the computed probabilistic results. These modules are integrated via the class endpoint, which connects all TOXTRUST classes and functions with the end user.

Functionally, TOXTRUST is divided into three modules responsible for handling individual pieces of toxicological evidence, combining them, and making decisions based on the computed probabilistic results. All three modules can be seen as sub-systems that bundle data and functionality together, operated through an “umbrella system” called *endpoint*, as shown in Fig. 7. Operationally, TOXTRUST consists of additional modules responsible for the configuration of the software on the local computer, management of the projects, and connection to the user interface.

#### 3.2.1 Endpoint

The endpoint class serves as the central component that enables and regulates the use of implemented features, ensuring the mathematical accuracy of the results. This class gathers input from the user, processes it by initialising other relevant classes or functions, and securely stores the data through local file permanence. Each operation is accompanied by a message informing the user of its success or failure.

#### 3.2.2 Single evidence

This class gathers and processes information that describes the result and the method used to produce it. Required variables include **identifier, source, result**, and **reliability. Relevance** and **weight** are optional variables that enhance the informational value of the evidence.

Each piece of evidence is assigned an identifier and classified into one of five source categories:

• “*In silico*”
• “*In vitro*”
• “*Expert*”
• “*Alert*”
• “Read across”

Results can be expressed in two ways, either as binary labels (0 or 1) or as probability values within the range [0, 1].

Reliability quantifies the performance of the body of evidence in producing the result. The tool supports a wide range of quantitative reliability metrics input as verbal prompts, ensuring maximum flexibility. Specificity and sensitivity are common measures for assessing toxicological methods. Whenever possible, it is recommended to consider prior information and update such scores to positive predictive values and negative predictive values (Bours 2021). Conversely, if the available data is relatively poor, more general measures such as accuracy can also be considered. Expert judgment can be considered as an alternative when statistical reliability metrics are unavailable.

Relevance assesses the appropriateness of an evidence piece for answering the assessment question. The distinction between reliability and relevance in toxicity assessments is outlined by Johnson et al. (2021). In TOXTRUST, relevance scores are offered as a dictionary of terms with corresponding numerical values:

• *Certain*: 1
• *Probable*: 0.9
• *Plausible*: 0.75
• *Equivocal*: 0.5
• *Doubted*: 0.25
• *Improbable*: 0.1
• *Impossible*: 0

Certain is the default score. However, adjustments may be made in cases when downgrading the relevance is necessary to better characterise the result. For example, an *in vitro* test might demonstrate high specificity, yet its negative result might still be ambiguous. By adjusting relevance, the new score is merged with the result and reliability during the computation of basic probability masses.

The weight variable can be used to rank the relative importance of evidence pieces among each other. In TOXTRUST, the reasoning behind the “weight of evidence” approach aligns closely with the definition provided by the OECD (2019): *“WoE can be generally understood to mean a method for decision-making that involves consideration of known lines of evidence where a “weight” is assigned to each line of evidence based on the confidence associated with the evidence.”* The default weight is 1, which can be replaced by any other integral number not larger than 5. For instance, an *in vitro* test can count double (weight = 2), as compared to an *in silico* model (weight = 1) predicting the same endpoint. As a result, the *in vitro test* result would be duplicated and treated as two separate pieces of evidence to be combined with the *in silico* model result.

For each piece of evidence, the class computes **basic probability masses** for the three elements of the power set: “Negative”, “Uncertain”, and “Positive”. These results are displayed in a tabular format. In addition, the degrees of Belief and Plausibility are also computed and stored for further analysis and visualisation.

#### 3.2.3 Evidence combination

The combination class serves to integrate the individually added evidence pieces using different DST-based rules and to visualise the final result. A set of inputs and user-defined settings is required to initialise this class. The most critical input is the **list of evidence pieces**, which must be pre-stored in the file system. Users should enable the "weight of evidence" option if weights were previously modified for the selected evidence pieces.

Next, the users can select one of three **combination rules** (Dempster’s, Yager’s, and Inagaki’s rule). For Inagaki’s rule, the scaling factor *c* can additionally be specified. To simplify this selection, numeric values for the scaling factor are accompanied by descriptive labels:

• *Full confidence*: 1
• *Partial confidence*: 0.75
• *Balance*: 0.5
• *Partial uncertainty*: 0.75
• *Full uncertainty: 0*

The default label for *c* is *balance*: 0.5.

To assist users who may have difficulties selecting the most appropriate combination rule, TOXTRUST offers an **“auto” rule-selection** option. Once activated, the process begins by retrieving the prestored data for each evidence piece specified for combination. Then, the auto rule-selection function analyses the characteristics of the provided evidence and points to the most appropriate rule based on different criteria, evaluated in two rounds of checks.

• Uncertainty check:

For each evidence piece, the function compares the uncertainty level to a user-defined maximum **uncertainty threshold**. If any evidence piece exceeds this threshold, the system selects Yager’s rule, which is suitable for handling high levels of uncertainty. If the uncertainty check passes (the uncertainties levels associated with all considered evidence pieces are within the predefined threshold), TOXTRUST performs a cross-evidence conflict check to evaluate the level of agreement among them.

• Conflict check:

An internal TOXTRUST variable called **maximum acceptable conflict** is calculated for each evidence piece as the average of its “Positive” and “Negative” degrees of Belief. If the Belief in “Negative” exceeds this threshold, the evidence is considered to support a negative outcome. Similarly, if the Belief in “Positive” exceeds it, the evidence favours a positive outcome.

After completing both checks, if all evidence pieces consistently point in the same direction (all outcomes are negative or positive), Dempster’s rule is chosen. Conversely, in cases of disagreement (some outcomes are negative, and some are positive) without exceeding the uncertainty threshold, Inagaki’s rule is applied with the default scaling factor *c*.

This logic can be summarised as follows:

1. *Yager’s rule:* if any evidence piece exceeds the uncertainty threshold
2. *Dempster’s rule*: when all evidence sources agree
3. *Inagaki’s rule*: in cases of significant conflict between evidence sources, with the default factor *c*

Whether the evidence combination is manual or automatic, the class computes **joint basic probability masses** for the three elements of the power set: “Negative”, “Uncertain”, and “Positive”.

These results are displayed in a tabular format and visualised through an **interactive diagram**, which provides an intuitive representation of the computed probabilities. Additionally, the degrees of Belief and Plausibility for the combined evidence are automatically calculated and stored with the results for further analysis.

#### 3.2.4 Decision maker

The decision maker feature in TOXTRUST assists users in making transparent decisions by incorporating settings defined before evidence collection or combination. These settings specify the maximum allowed level of uncertainty (**uncertainty threshold**) associated with a specific outcome and the minimum required level of Belief (**confidence threshold**) in that outcome.

When applying the decision maker to a particular evidence piece, the function first examines whether the uncertainty threshold is not exceeded. If this condition is not fulfilled, the decision falls on the “Uncertain” element. In an opposite case, the analysis is complemented by comparing the degrees of Belief for both possible outcomes (“Negative” and “Positive) against the confidence threshold. The final decision falls on the outcome lying above the confidence threshold.

The decision maker feature is designed to operate flexibly for both classes, single evidence and evidence combination. The resulting decisions can be presented either as **verbal outputs**, pointing to one of the three possible options (“Negative”, “Uncertain”, or “Positive”) or through **error diagrams**, which visually depict the bounds of Belief and Plausibility.

## 4 TOXTRUST user interface

The GUI of TOXTRUST is designed to run on any computer on a local server, accessible through the browser at a host address (http://localhost:5000). After starting the server, the welcome page (Supplementary Fig. 1) features a brief description of the tool in the centre and an interactive drop-down menu in the upper left corner. This menu provides the essential functions, including creating new projects, accessing existing projects, viewing documentation, navigating to the code repository, and consulting references. The design, with the colourful graphic at the top and the white placeholder space for user input, is uniformly applied across all sections of TOXTRUST.

### 4.1 New project

Creating a new project in TOXTRUST is straightforward (Supplementary Fig. 2). After selecting the corresponding option from the main menu, a new window appears, prompting the user to provide a name for the project (e.g., *Mechanism X*). In TOXTRUST, all blue buttons symbolise additional functions or features. When a new project is created, the tool creates a placeholder to ensure file permanence for that project, which the interface automatically advances to the next step.

### 4.2 Endpoint

Each project in TOXTRUST is organised into four consecutive steps, which are displayed at the top of the dialogue. The first step (Supplementary Fig. 3) involves providing general information about the endpoint, such as the name of the compound, testing framework, a description of the assessment question, and project permissions reflecting its confidentiality status. Once the required information is entered, it can be saved.

### 4.3 Settings

The second project step (Supplementary Fig. 4) requires the user to specify their preferences to guide decision-making and evidence combination. In the *Decision Settings* section, users must define both the maximum allowable level of uncertainty and the minimum required level of belief. These are explained in Section 3.2.4 as the uncertainty and confidence thresholds, respectively. In the *Combination Settings* section, users can either manually select a rule or tick the “Auto rule selection” option. If the latter is chosen, the tool disables further rule settings, as they become irrelevant. At this stage, users can also decide whether to combine evidence following the weight of evidence concept. The combination options are extensively described in Section 3.2.3.

### 4.4 Evidence

In the third project step, evidence pieces are collected. The “Add evidence” dialogue (Supplementary Fig. 5) displays all required (identifier, source, result, reliability) and optional variables (relevance, weight), with the corresponding options broken down in Section 3.2.2. In the result section, users can tick either one of the outcomes (“Negative” or “Positive”) or both.

• If only one outcome is selected (e.g., to indicate a result of an *in vitro* experiment), probability values are not required.
• If both outcomes are selected (e.g., to consider predicted probabilities from *in silico* models), the probability box becomes mandatory, and the sum of the provided probabilities must equal 1.

The reliability dialogue is directly linked with the result section. Here, users can specify the metric verbally and add the corresponding value.

Once a new evidence piece is saved, the data provided by the user—along with the computed probabilities for all possible outcomes (“Negative”, “Uncertain”, “Positive”) and the decision, are displayed in a table (Supplementary Fig. 6). Additional features include a *Combine* column for selecting evidence pieces for combination and a *Delete* column for easy removal of individual entries.

Clicking on the blue-marked outcome (e.g., “Negative”) in the *Decision* column opens an additional window (Supplementary Fig. 7) divided into two sections. The upper section displays a table with the computed bounds for the degrees of Belief and Plausibility for the outcomes “Negative” and “Positive”. The lower section contains a plot showing probability bounds only for outcomes with non-zero results. The function of the latter is to visually inform users about the level of uncertainty associated with the given evidence piece and provide a foundation explaining the decision made.

### 4.5 Combination

After selecting evidence pieces in Step 3 and clicking the *Combine* button, the data are integrated considering the settings submitted in Step 2. As for single evidence, also for their combination, the relevant data, the computed probabilities for all possible outcomes (“Negative”, “Uncertain”, “Positive”) and the decision are shown in a table (Supplementary Fig. 8). Regarding the relevant data, they cover the endpoint name, the list of combined single evidence pieces, and the combination rule. The decision feature is designed in the same way as described above. The combination result can be easily removed by clicking the *Delete* button.

### 4.6 Existing projects

Projects in TOXTRUST are managed through file permanence and can be accessed anytime from the main menu, regardless of their stage of advancement (Supplementary Fig. 9). Each time a new project is created, TOXTRUST generates a subfolder in the local file system to store all associated data, ensuring its availability for later retrieval. The *Existing projects* tab displays a table listing the general information about the endpoints collected in Step 1, including the endpoint name, compound, testing framework, and project permissions. Projects can be easily removed by clicking the *Delete* button.

### 4.7 Practical use example

#### 4.7.1 Background information

Before their commercialization, chemicals included in the formulations of personal care products, pesticides, and cleaning supplies must be tested to determine whether they may cause irritation or other acute toxicity effects in case of eye contact. In March 2019 the Scientific Committee on Consumer Safety (SCCS) issued its final opinion supporting the safe use of Ethylzingerone as a preservative in rinse-off, oral care, and leave-on cosmetic products to concentration of up to 0.7% (Bernauer et al. 2019). Ethylzingerone, also known as Hydroxyethoxyphenyl Butanone (HEPB), is registered under Cosmetics Europe No P98 and CAS No 569646-79-3.

The SCCS based its decision on two dossier submissions. The first concluded that Ethylzingerone is generally safe at the specified maximum concentration. The second included additional data generated for the specific purpose of re-evaluating the potential of Ethylzingerone to cause eye irritation. Results from the Reconstructed Human Cornea-Like Epithelium (RhCE) Test Method (SkinEthic^TM^ HCE model) resolved the eye safety concerns and confirmed no need for classification under the UN GHS (“No Category”).

### 4.8 Case study description

**Fig. 8:**
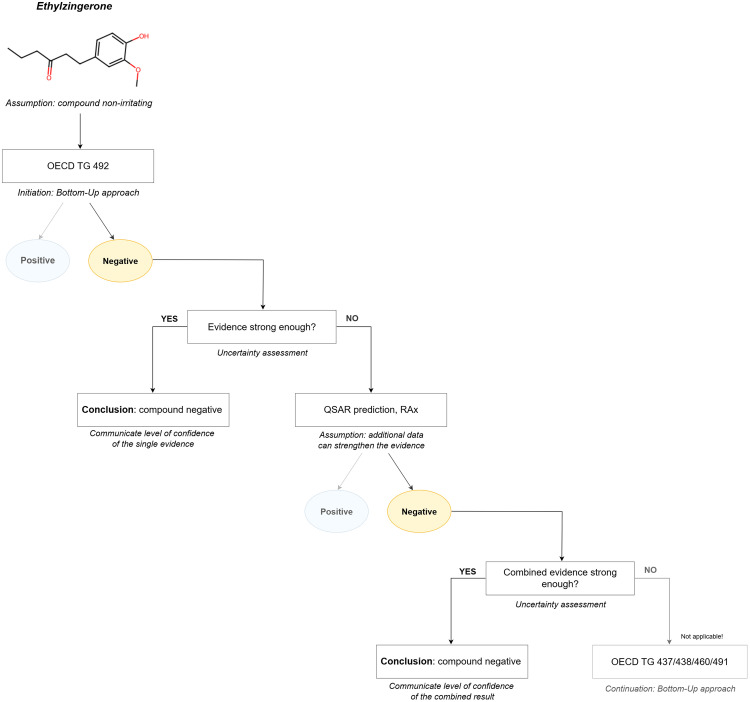
A hypothetical case study workflow based on a real assessment focusing on the potential of Ethylzingerone to cause eye irritation. The workflow begins with the assumption that Ethylzingerone is non-irritating to the eyes, based on a negative result from the RhCE test (OECD TG 492). The DST framework is then applied to assess the uncertainty associated with this RhCE test result and combine it with a QSAR model prediction and a RAx-based expert judgment.

Using this real assessment case as a basis, we developed a fully hypothetical case study workflow, shown in Fig. 8*,* which starts with an initial assumption that Ethylzingerone is non-irritating to the eyes. Given the initial assumption and following a Bottom-Up testing approach described in OECD TG 492 (OECD 2025), the negative result obtained from the RhCE test model was selected as the central piece of evidence.

This case study first illustrates how DST can be applied to quantify the levels of uncertainty associated with an *in vitro* result. If the result is negative and the uncertainty is low, the expert may decide to accept the initial assumption. However, if the result is negative but the uncertainty exceeds an acceptable threshold, the expert may choose to collect additional evidence from alternative sources.

Second, this case study aims to demonstrate how DST-based combination rules can be applied to integrate multiple lines of evidence, including an *in silico* Quantitative Structure-Activity Relationship (QSAR) model prediction and a Read-Across (RAx) based expert judgment.

To encode this case study in TOXTRUST, the steps described in Sections 4.1 – 4.2 were followed. The assessment question was defined as the one in the published SCCS opinion: “*In light of the new studies provided, does the SCCS consider the use of Hydroxyethoxyphenyl Butanone (HEPB) safe with regard to eye irritation, when used as preservative in rinse-off, oral care and leave-on cosmetic products with a maximum concentration of 0.7%?”* As the RhCE test does not distinguish between the potency of irritation, the frame of discernment contains two elements: “safe” (non-irritating) and “unsafe” (irritating), which can be translated to “Negative” *(N)* and “Positive” *(P)*, respectively. The decision settings were kept at their default values, as explained in Section 4.3.

### 4.9 Single evidence

The RhCE test produced a negative result for Ethylzingerone. The most suitable document for quantifying the reliability of this experimental method is the Validation Study Report on “The EURL ECVAM - Cosmetics Europe prospective validation study of Reconstructed human Cornea-like Epithelium (RhCE)-based test methods for identifying chemicals not requiring classification and labelling for serious eye damage/eye irritation” by Barroso et al., 2015.

The specificity values were computed for two thresholds of mean cell viability (50% and 60%), which serve to distinguish compounds that require classification from those that do not, as per the UN GHS. Since the measurements of cell viability exceeded both thresholds, no specification was required. For our example, we select 50% as the mean viability threshold to 50% and read off the reported specificity value of 64.3% (≈ 0.64).

The experimental result and reliability metric were documented in TOXTRUST as described in Section 4.4. Relevance was defined as “Certain” and although not yet required at this step, the weight was specified as 2. TOXTRUST produced results shown in Table 1.

**Table 1:** Basic probability masses computed for the RhCE test outcome.

Outcome	Negative	Uncertain	Positive
Basic probability mass	0.64	0.36	0

The outcome generated by a NAM (the “Negative” result) and the overall performance of the NAM in generating that outcome (the specificity value of 0.64) were integrated, yielding a basic probability mass of 0.64 assigned to the “Negative” (non-irritating) outcome. The remaining 0.36 reflects the uncertainty related to this negative result. The probability mass for the “Positive” (irritating) outcome is zero.

Considering the definition of Belief introduced in Section 2.9, defined as the probability fully related to a specific outcome, the Belief associated with the “Negative” outcome is also 0.64. The scientific conclusion based on this result could be formulated by indicating that the decision is negative, with the precise probability for this outcome lying within the lower and upper probability bounds of [0.64–1.00]. Given that the maximum uncertainty allowed in TOXTRUST settings for decision making was set to 0.3, this result exceeds that threshold and was therefore labelled as “Uncertain” after applying the DST.

### 4.10 Additional evidence

Although, according to the UN GHS, increasing the number of the data sources is not necessary for hazard classification of eye irritation, it can be helpful. In an ideal scenario, a QSAR model would be chosen for classifying irritating and non-irritating compounds, trained using data generated by the RhCE test, specifically the SkinEthic™ HCE model. The model should be validated according to the “OECD Principles for the Validation, for Regulatory Purpose, of (Q)SAR Models”(OECD 2004). The numerical values used in his example were not real and served only for illustrative purposes. Information on its predictive performance, assessed through external validation, reported sensitivity and specificity values of 0.84 and 0.88, respectively. The predicted probabilities from this model were 0.76 for the “Negative” (non-irritating) class and 0.24 for the “Positive” (irritating) class. The weight and relevance were set to 1 and “Plausible,” respectively.

The third body of evidence in this case study is the RAx-based expert judgment. We assume the expert followed the principles outlined in the Read-Across Assessment Framework (RAAF) by ECHA (2017). During knowledge elicitation, they proposed assigning “No Category” and estimated a 90% probability that Ethylzingerone would not cause eye irritation, assigning the remaining 10% to the opposing outcome. These results can be expressed as 0.9 (“Negative”) and 0.1 (“Positive”). Furthermore, the expert estimated the reliability of their judgment as 90% and considered the relevance of the RAx data as “Probable.” The weight of this assessment was set to 1.

All data selected for combination are summarised in Table 2.

**Table 2:** Overview of evidence lines selected for combination.

Name	Type	Weight	Relevance	Result (Reliability)	
				**Negative**	**Positive**
RhCE	In vitro	2	Certain	1 (0.64)	N/A
QSAR	In silico	1	Plausible	0.76 (0.84)	0.24 (0.88)
RAx	Read-Across	1	Probable	0.9 (0.9)	0.1 (0.9)

### 4.11 Evidence combination

Results of the evidence combination generated by TOXTRUST are shown in Table 3. The three different combination methods explained in Section 2.8 were applied to illustrate their differences and provide guidance on interpreting the results.

**Table 3:** Joint basic probability masses computed using diverse rules for the provided evidence.

Rule	Negative	Uncertain	Positive
Dempster’s	0.94	0.03	0.03
Yager’s	0.76	0.22	0.02
Inagaki’s	0.85	0.12	0.03

After combining evidence using Dempster’s rule, the joint basic probability mass and the Belief in the “Negative” outcome increased to 0.94. While the individual *in vitro* RhCE test was labelled as “Uncertain” after applying DST, the combined result can be labelled as “Negative” with very high certainty. The scientific conclusion based on this combination could be formulated as negative, with the probability for this outcome falling within the lower and upper bounds of [0.96–0.99]. Incorporating more evidence sources not only strengthened the negative *in vitro* RhCE result but also reduced the associated uncertainty from 0.36 to 0.03.

A key difference between Dempster’s and Yager’s results is the higher joint basic probability mass associated with the “Uncertain” element. This is because Yager’s rule handles uncertainty more rigorously than Dempster’s rule, attributing the degree of conflict to the level of uncertainty, instead of normalising the joint basic probability masses. The probability bounds for this outcome is between [0.76–0.98]. Although the conclusion still supports the negative result, the presence of more conflicting evidence under Yager’s approach could lead to a less decisive outcome, potentially leaving the initial assumption unconfirmed.

When comparing the joint basic probability masses among these three combination results, the uncertainty approximately reaches a midpoint when applying Inagaki’s rule with factor c set to the TOXTRUST default of 0.5. This observation is meaningful because the scaling factor serves to establish a balance between rigor and decisiveness. The conclusion regarding the safety of Ethylzingerone concerning eye irritation was reconfirmed using Inagaki’s combination rule. The degree of Belief associated with this conclusion, based on the combination result, is 0.85. Adding the ignorance, the probability bounds of Belief and Plausibility span the interval [0.85–0.97].

## 5 Discussion

This article illustrates that the Dempster-Shafer Theory satisfies two critical requirements in modern toxicology: combining evidence and reasoning with uncertainty. Toxicologists are becoming increasingly familiar with the DST, as it is frequently featured in emerging uncertainty assessment frameworks, which integrate various probabilistic and mathematical methods (Sahlin and Tang 2024). However, the practical application of DST is challenging due to its high level of abstraction and the complexity introduced by numerous parameters, equations, and requirements.

Moreover, as the universe of NAMs continues to expand and NGRA frameworks become progressively more complex, policymakers continue to raise expectations for uncertainty analysis, probabilistic problem-solving, and an unbiased combination of evidence (van der Zalm et al. 2022). These requirements highlight the demand for *in silico* solutions that ensure mathematical correctness, objectivity, and reproducibility.

To address these challenges, we developed TOXTRUST, an open-source software designed to assist experts in applying DST to solve specific toxicological problems. This development marks a significant milestone, addressing a major gap in toxicology—the limited availability of computational tools that simplify complex mathematical procedures and support expert decision-making. Beyond that, we aim to set an example of how theoretical mathematical approaches can be transformed into user-friendly toxicological applications. Our goal is to advance the field by moving beyond traditional risk assessment methods and the reliance on textual documentation of results.

Focusing on the design, TOXTRUST is a Python-based library integrated into a visually appealing and user-friendly GUI. The GUI is operated through a drop-down menu with essential functions such as creating new projects, accessing previous projects, viewing documentation, navigating to the code repository, and consulting references. A new project is completed in four consecutive steps: (1) entering a generic description of the endpoint, (2) configuring settings, (3) adding evidence pieces, and (4) combining them. After each evidence piece is added in step 3, the GUI displays the user input alongside the computed probabilities for three possible outcomes: “Negative,” “Uncertain,” and “Positive” as well as the corresponding decision. This decision includes an expandable option to show a detailed table of the degrees of Belief and Plausibility, accompanied by a graphical representation of probability bounds. In step 4, the combination tab tabularly summarises the computed probabilities and a decision, using a similar approach. This table is accompanied by an interactive graphic that illustrates both the individual evidence pieces and their combination result. All plots generated by TOXTRUST are easily downloadable for documentation. Overall, the GUI is relatively simple yet contains all the features required to effectively apply the DST to a toxicological problem.

From the functional aspect, TOXTRUST can currently handle any kind of endpoint with binary end-results. However, considering that toxicological decisions often involve grouping, the expansion of applicability from binary to categorical variables is an important ongoing process. Regarding the implemented functionalities, the mathematical foundation of the tool rests on the original theory. However, expanding the system by adding novel methods for, for instance, the basic probability assignment (Tang et al., 2023), conflict handling (Yan et al. 2022) combination of evidence (Xue et al. 2021; Sarabi-Jamab and Araabi 2018), an uncertainty estimation (Sarabi-Jamab and Araabi 2020) would additionally increase its innovation status. Moreover, the quality of decision systems is often defined by the quality of the visualisation of input and output data. In that sense, implementing additional visual mapping strategies, such as those described by Weiskopf (2022) could enhance the communication of uncertainty to the parties involved in decision-making. As TOXTRUST is under constant development, these and several other features will be included in future releases.

But, while stand-alone *in silico* methods are very useful, integrated informatics systems offer universal solutions to a wide range of problems. Therefore, we envision the inclusion of TOXTRUST within the knowledge and method management platforms created for large-scale projects supporting the NGRA, for instance, RISK-HUNT3R (Pallocca et al. 2022), ONTOX (Vinken et al., 2021), PrecisionTox (PrecisionTox, Consortium., 2023), belonging to the ASPIS cluster. Such integration would allow for an immediate and correct use of the tool for clearly defined purposes while restricting its use where not applicable.

These projects are linked through the joint efforts to develop the ASPIS Safety Profiling Algorithm (ASPA), based on a tiered approach for the NGRA in the risk assessment of chronic adverse health effects (EC 2023). ASPA logically orders several analytical tasks and expert decisions that form an essential part of the NGRA. Computationally, the ASPA workflow was encoded within NAMASTOX (Pastor 2022), a computational tool with a graphical interface that can guide the users in the step-by-step application of the workflow, indicating “what to do next”, suggesting the most appropriate NAMs, and collecting the NAM results in a systematic and ordered way. The flexibility of this interface allows for a smooth integration of novel uncertainty analysis methods and sophisticated probabilistic approaches developed for specific steps of the ASPA workflow. In that context, the incorporation of TOXTRUST is planned for NAMASTOX as part of the set of features supporting the characterisation, quantification, and combination of uncertainty for various types of NAM results.

In the NGRA, case studies play a pivotal role in assessing and applying new approaches, whether they serve for data generation or integration (Westmoreland et al. 2022; ME et al. 2011). Hence, to assess the true value of our tool, we plan to involve stakeholders from regulatory agencies, industry representatives or academia to process materials provided in publicly available case studies and to apply TOXTRUST to handle specific problems within these. This review and refinement process could be further enhanced through the application of the artificial intelligence-based concept of e-validation, introduced by Hartung, Maertens, and Luechtefeld (2024).

We believe that the methodology described in this article would help experts acknowledge the presence of uncertainty and increase the transparency of toxicological decisions.

## Ethical standards

The manuscript does not contain data from animal studies, clinical studies or patient data.

### Data and materials availability statement

TOXTRUST source code is available at GitHub under GNU GLP-3.0 license at the following repositories:

• Backend: https://github.com/phi-grib/TOXTRUST
• API (web server): https://github.com/phi-grib/TOXTRUST_api
• WEB (user interface): https://github.com/phi-grib/TOXTRUST_web

No dataset was described nor required to support the conclusions of the manuscript.

Operating system(s): Platform independent and tested in Windows, Linux, and iOS.

Programming language: Python, Typescript (Angular).

Other requirements: TOXTRUST uses an Anaconda environment defining the required Python libraries

Graphics explaining TOXTRUST were generated using icons downloaded from https://www.flaticon.com/

## CRediT authorship contribution statement

**Karolina Kopańska:** Writing – review & editing, Writing – original draft, Visualization, Validation, Software, Methodology, Conceptualization. **Adrian Cabrera:** Visualization, Software, Methodology. **Manuel Pastor:** Writing – review & editing, Supervision, Resources, Project administration, Methodology, Investigation, Funding acquisition, Conceptualization.

## Declaration of competing interest

The authors declare that they have no known competing financial interests or personal relationships that could have appeared to influence the work reported in this paper.

## Data Availability

The authors used one result which is publically available and appropriately cited in this manuscript.

## Appendix Supplementary materials

Image, application 1
