# Supplementary material for: TOXTRUST: a tool leveraging the Dempster-Shafer Theory for robust integration of NAM results in decision-making considering uncertainty
Source: NAM J. 2025 Aug 10;1:100043. doi: 10.1016/j.namjnl.2025.100043 (PMC13289040; doi:10.1016/j.namjnl.2025.100043)
Supplement: Supplementary file 1 [file mmc1.docx]

**Title**

TOXTRUST: a tool leveraging the Dempster-Shafer Theory for robust integration of NAM results in decision-making considering uncertainty

**Author information**

Karolina Kopańska*^1*, 2^*, Adrian Cabrera*^1^*, Manuel Pastor*^1*^*

*^1^ Research Programme on Biomedical Informatics (GRIB), Department of Medicine and Life Sciences, Universitat Pompeu Fabra, Hospital del Mar Research Institute, Barcelona, Spain*

*^2^* *Center for Alternatives to Animal Testing (CAAT), Johns Hopkins University, Baltimore, USA*

Corresponding author: Manuel Pastor ([manuel.pastor@upf.edu](mailto:manuel.pastor@upf.edu))

*Main affiliation where this work was carried out

**Supplementary materials – TOXTRUST installation guide**

1. **Download and install Anaconda**

Anaconda is a software distribution of Python for scientific computing, allowing to define the essential libraries, facilitating their installation, and keeping track of package versions to avoid incompatibilities.

A detailed installation guide for Windows / MacOS / Linux can be found under the following link: <https://docs.anaconda.com/anaconda/install/>

1. **Overview of TOXTRUST repositories**

The source code of the backend and frontend of TOXTRUST is stored in GitHub:

- Backend: <https://github.com/phi-grib/TOXTRUST>
- API (web server): <https://github.com/phi-grib/TOXTRUST_api>
- WEB (user interface): <https://github.com/phi-grib/TOXTRUST_web>

Both, the API and WEB are required to make the graphical user interface run on a server. These two code elements were used separately during the development of TOXTRUST and can be used if development mode is preferred.

However, to allow running TOXTRUST in user mode, the user interface (WEB) was compiled and introduced into the web server (API). Hence, in user mode, a separate starting of the WEB is not required.

1. **Installation of TOXTRUST Python package**

Before initiating the download, it recommended to create a new folder within the local file system where all TOXTRUST repositories will be stored.

- 1. Once created, navigate to the desired folder within an anaconda terminal and clone the repository

*git clone* <https://github.com/phi-grib/TOXTRUST.git>

- 1. Enter TOXTRUST repository via terminal

*cd TOXTRUST*

- 1. Create "toxtrust" anaconda environment with all the dependencies

*conda env create -f environment.yml*

- 1. Once the environment is created, activate it.

*conda activate toxtrust*

*4.5. TOXTRUST must be installed as a regular Python package.*

*For user mode only, execute (note the dot at the end)*

*pip install .*

*For* development mode*, execute (note the dot at the end)*

*pip install -e .*

1. **Installation of TOXTRUST user interface**
   1. Using a new terminal window, navigate anaconda terminal to the desired folder where the interface should be installed and clone the repository

*git clone* <https://github.com/phi-grib/TOXTRUST_api.git>

- 1. Enter TOXTRUST_api repository via anaconda terminal

*cd TOXTRUST_api*

- 1. Activate toxtrust environment

*conda activate toxtrust*

- 1. Run the interface

*python app.py*

- 1. Run TOXTRUST local server via web browser (e.g., chrome)

[*http://localhost:5000/*](http://localhost:5000/)

**Supplementary figures**

The supplementary figures complement Section “4 TOXTRUST user interface”.


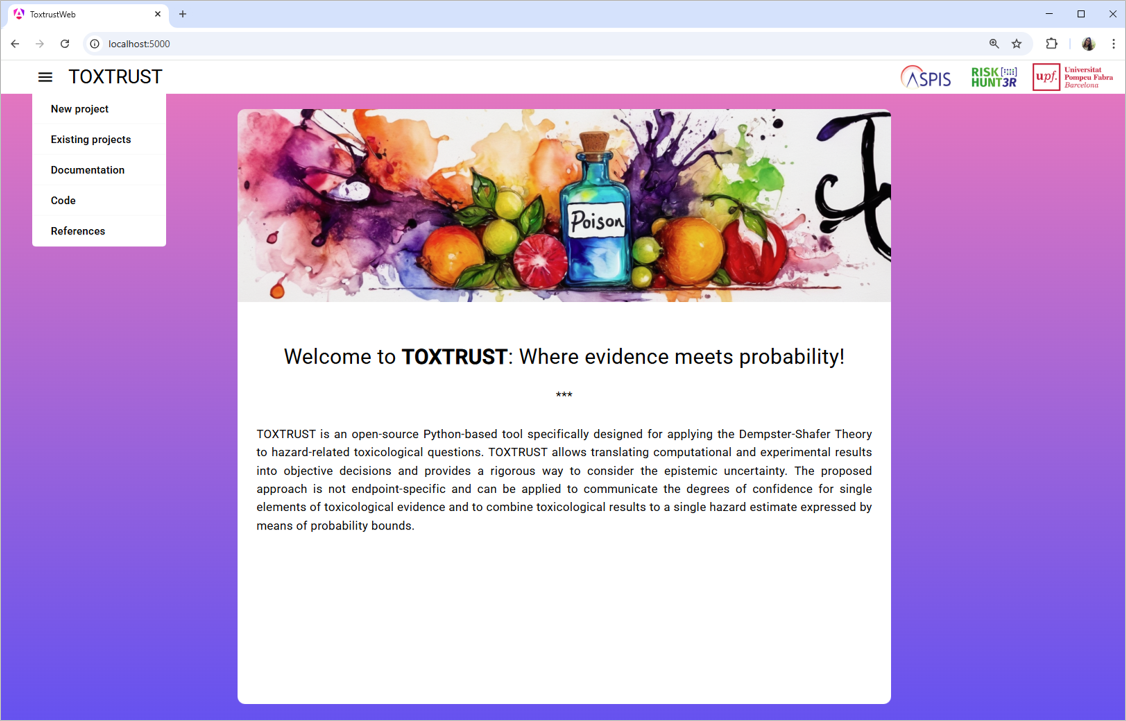


***Supplementary Fig. 1***: Welcome page of TOXTRUST, featuring a brief description of the tool in the centre and an interactive drop-down menu in the upper left corner.


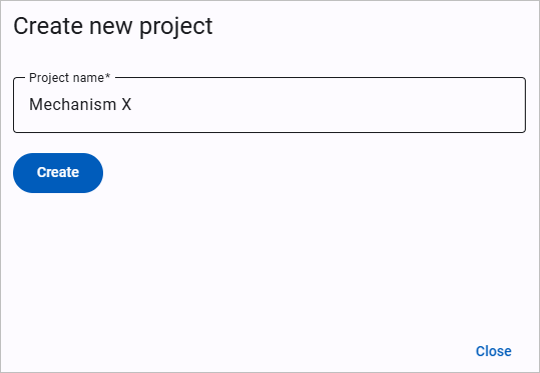


***Supplementary Fig. 2***: After selecting the “New project” option from the main menu, a new window appears, prompting the user to provide a name for the project.


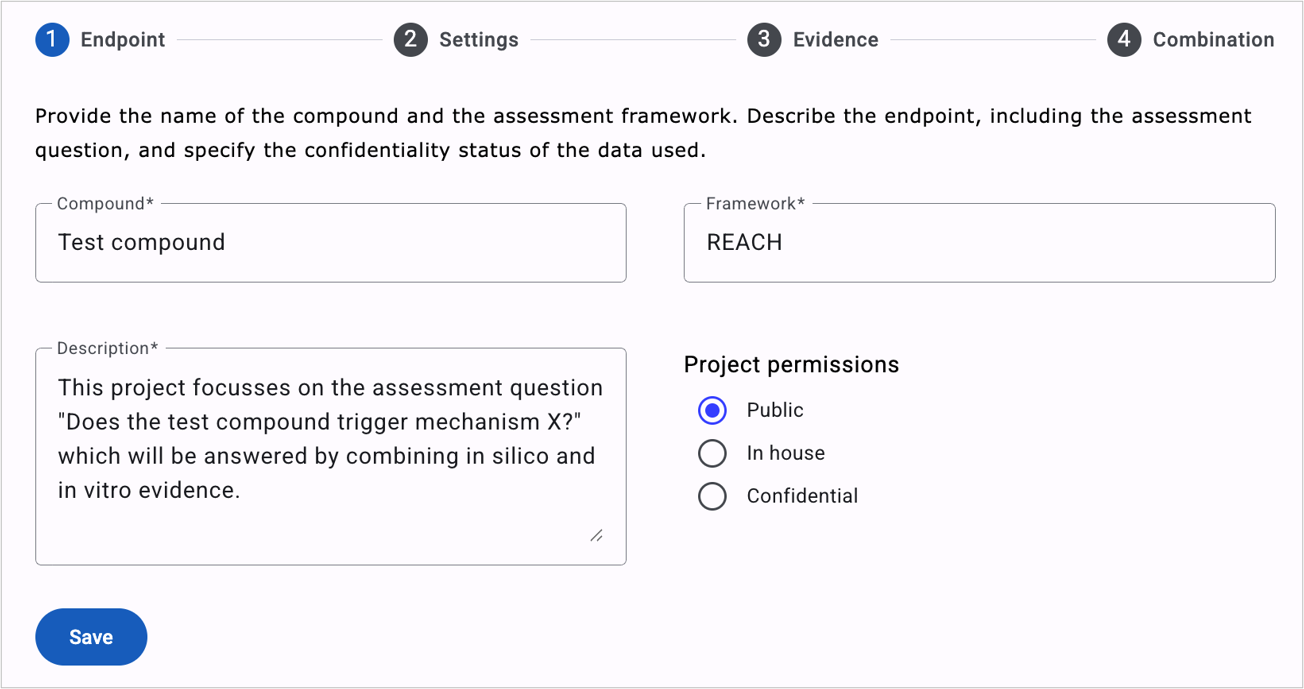


***Supplementary Fig. 3***: In TOXTRUST, projects are organised into four consecutive steps, which are displayed at the top of the dialogue. The first step involves characterising the new project by providing information about the endpoint.


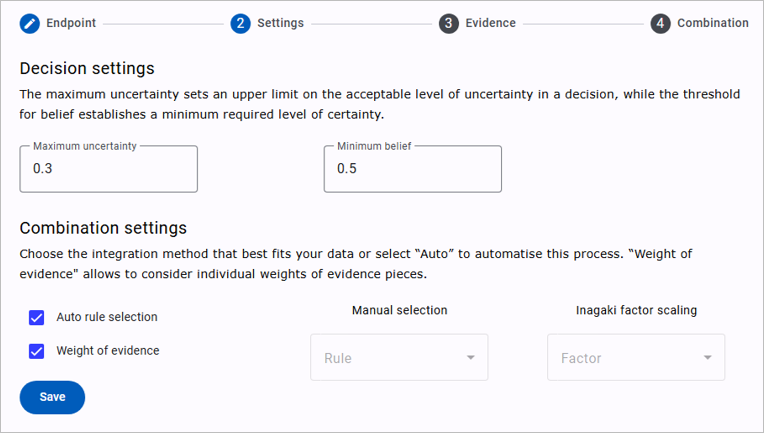


***Supplementary Fig. 4***: The second project step prompts users to specify settings for decision-making and evidence combination.

***
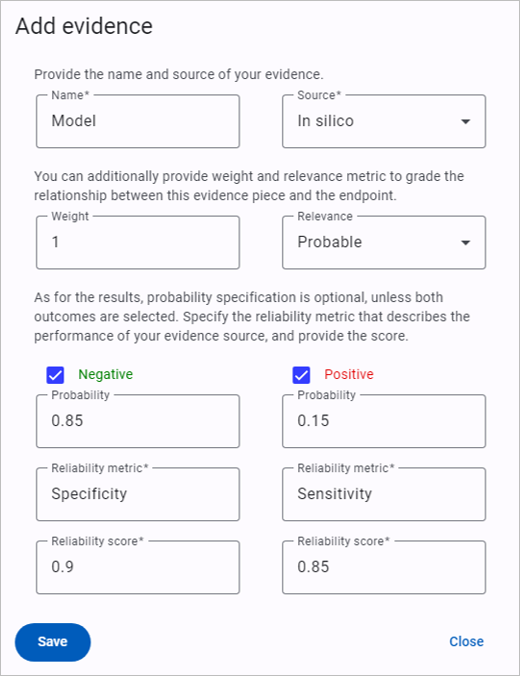
***

***Supplementary Fig. 5***: The “Add evidence” dialogue consists of all required (identifier, source, result, reliability) and optional variables (relevance, weight) characterising a new evidence piece.


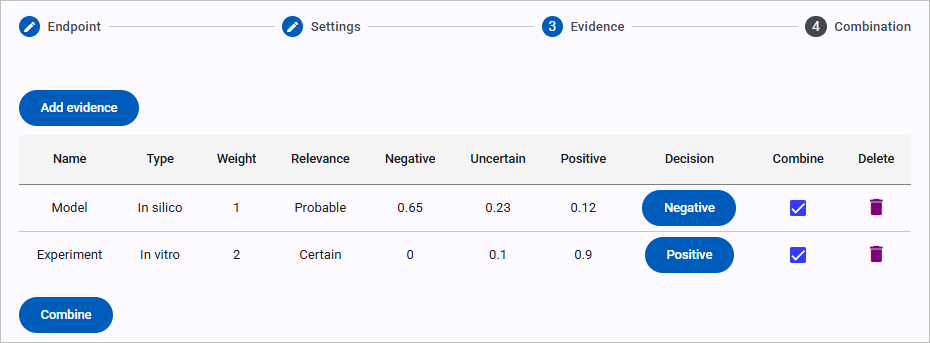


***Supplementary Fig. 6***: Individual evidence pieces are displayed in a table with the data provided by the user, the computed probabilities for all possible outcomes (“Negative”, “Uncertain”, “Positive”), and the decision. The last two columns serve to select evidence pieces for combination or to remove them.


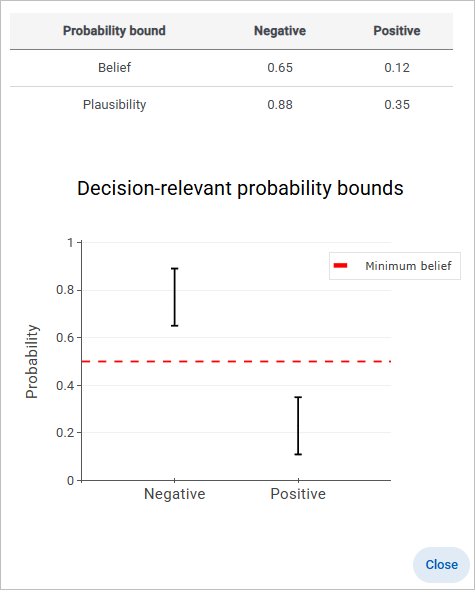


***Supplementary Fig. 7***: The decision features of TOXTRUST are extended to an additional window divided into two sections. The upper section displays a table with the computed bounds for the degrees of Belief and Plausibility for the outcomes “Negative” and “Positive” and the lower section contains a plot that illustrates these bounds for outcomes with non-zero results.


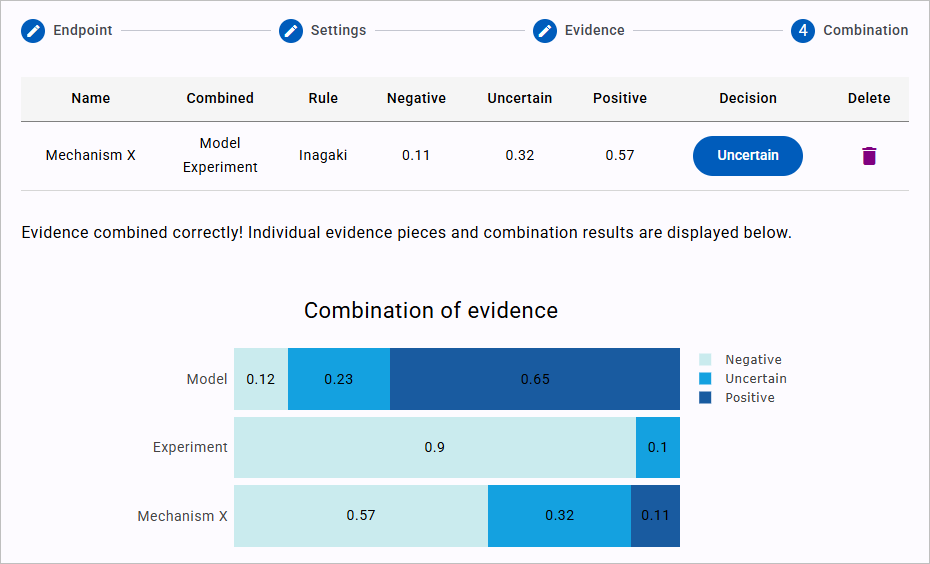


***Supplementary Fig. 8***: The Combination tab contains a table with relevant data, the computed probabilities for all possible outcomes (“Negative”, “Uncertain”, “Positive”), and the decision. Below, the computed probabilities are visualised employing a bar plot.


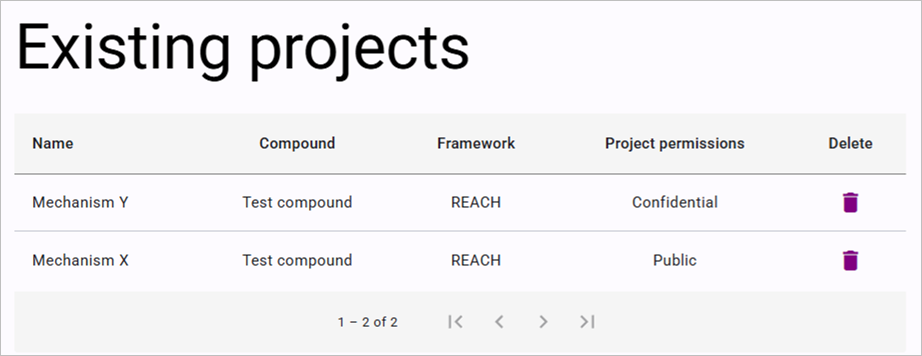


***Supplementary Fig. 9***: The *Existing projects* tab displays a table listing the general information about the endpoints including the project name, compound, testing framework, and project permissions.
